# Prescription Department

**Published:** 1895-07

**Authors:** 


CORNS.

Trim your corn in the gray of the morn,
With a blade that's shaved the dead,
And barefoot go, and hide it so
The rain will rust it red;
Dip your foot in the dew, and put
A print of it on the floor,
And stew the fat of a brindle cat,
And say this o'er and o'er:—
Corney! morny! bladey! dead!
Gorey! sorey! rusty! red!
Footsy! putsy! floory! stew!
Fatsy! catsy!
Mew!
Mew!
Come, grease my corn
In the gray of the morn!
Mew! mew! mew!
—JAMES WHITCOMB RILEY.

FOR CHRONIC ECZEMA OF THE FACE.

This is a modification of Hebra's ointment:

℞. Compound stearate of zinc, c. ichthyol....20 parts.
Acid salicylic......... 5 "
Glycerin.... ..........10 "
Albolene (white).......50 "

To be smeared over the affected part.

CATARRH, NASAL AND FAUCIAL.

℞. Acidi carbolici.............℈j.

Sodii boratis...............ʒj.

Sodii bicarbonatis..........ʒj.

Glycerinæ.................℥j.

Aq. rosæ..................℥j.

Aquæ.............. ......ad Oj.

M. Sig.: Use as a spray. (Nasal form).—LEFFERTS.

### EARACHE.

℞. Morph. muriat...........grs. v.

Atropin sulph............grs. i.

Ol olivæ.................ʒ i.

Glycerinæ. .. ...........ʒ iss.

M. S. gtt. iii-v in ear, and retain with cotton.

Repeat every hour.

Or, ℞. Camphor. chloral..m. v.
Glycerine..........xxxiii.
Almond oil........m. xxx.

M. S.—Three drops of this mixture, on absorbent cotton, to be placed in the ear twice a day.

—*Medical Progress.*

### PAINLESS TOOTH EXTRACTING.

The following has been found of value by many dentists:

℞. Ol. gaultheriæ.... ..drams ii.

Chloroform..... . .dram i.

Ether sulph.........dram i.

Chloralis ...........drams ii.

Ol. carophyll. .... .drams iv.

Alcohol..... ......drams xii.

M. S.—Apply with cotton pressed on each side of tooth.

—*Medical Standard.*

### IODINE MIXTURE NOT PRODUCING IODISM.

(Dr. Hardaway.—Med. Week, '94, p. 491.)

℞. Potassium iodide....oz. ½ to 1.

Iron and amm. citrate....dr. 2.

Tr. nux vomica........fl. dr. 2.

Water.............. .....fl. oz. 1½.

Tr. cinchona compound.fl. oz 2.

Teaspoonful in water after meals.

—*Amer. Med.- Surg. Bulletin.*

### INFANTILE ENTERO-COLITIS.

With hyperacidity of feces and pain with the motions:

℞. Sodii Bicarb............ ʒij.

Bismuth Subnit......... ʒiij.

Syrup. Simp............. ℥ss.

Aq. Cinnamon........... ℥jss.

M. S. Teaspoonful every two or three hours.

—*Louisville Medical Monthly.*

### CANCER.

℞. Zinci chlor...............ʒ ij.

Pulv. rad. althææ.... ...ʒ vj.

Aq. destillat..............q. s.

M. Et. ft. magma. Sig.: Apply to affected part. (In epithelioma).

—CANQUOIN.

